# Moving beyond symptom subtypes: testing a common dimension of lifetime OCD symptoms

**DOI:** 10.1017/S1092852925100679

**Published:** 2025-11-03

**Authors:** Abel S. Mathew, Sarah L. Garnaat, David R. Strong, Nicole C. R. McLaughlin, Kathleen D. Askland, O. Joseph Bienvenu, Janice Krasnow, Marco A. Grados, Bernadette Cullen, Fernando S. Goes, Steven Rasmussen, James A. Knowles, James T. McCracken, John Piacentini, Daniel Geller, Evelyn Stewart, Mark A. Riddle, Paul Nestadt, Gerald Nestadt, Jack Samuels, Benjamin D. Greenberg

**Affiliations:** 1 Department of Psychiatry and Human Behavior, Brown Medical School, Butler Hospital, Providence, RI, USA; 2 Division of Psychology, Department of Psychiatry, UT Southwestern Medical Center, Dallas, TX, USA; 3 Price, Proctor & Associates, Dallas, TX, USA; 4 Department of Psychiatry, Darmouth Geisel School of Medicine, Hanover, NH, USA; 5 Department of Family Medicine and Public Health, University of California San Diego, San Diego, CA, USA; 6 Askland Medicine Professional Corporation & Department of Psychiatry and Behavioural Neurosciences, McMaster University, Hamilton, ON, Canada; 7 Department of Psychiatry and Behavioral Sciences, Johns Hopkins University School of Medicine, Baltimore, MD, USA; 8 Department of Genetics, Rutgers University-New Brunswick, New Brunswick, NJ, USA; 9 Department of Psychiatry and Biobehavioral Sciences, University of California, Los Angeles, School of Medicine, Los Angeles, CA, USA; 10 Department of Psychiatry, Harvard Medical School, Boston, MA, USA; 11 Department of Psychiatry, Faculty of Medicine, University of British Columbia, Vancouver, BC, USA; 12 BC Children’s Hospital Research Institute, Vancouver, and BC Mental Health & Substance Use Services, Vancouver, BC, USA

**Keywords:** Obsessive-compulsive disorder, item response theory, Yale-Brown Obessive Compulsive Scale, lifetime OCD symptoms, Y-BOCS, OCD

## Abstract

**Introduction:**

Obsessive–compulsive disorder (OCD) is a neuropsychiatric disorder characterized by recurrent intrusive thoughts and ritualized behaviors, often aimed at reducing distress. OCD is heterogeneous in its presentation and many patients with OCD experience a variety of different symptoms throughout their course of illness. Efforts to understand symptom domains in OCD have typically identified three to five symptom domains, such as the domains of doubt/checking, contamination, superstitions/rituals, symmetry/hoarding, and taboo thoughts. Recent studies in the genetics of OCD have suggested a common OCD dimension may provide additional information above and beyond the previously identified symptom domains. Thus, we sought to test a hierarchical model of lifetime OCD symptoms and evaluate the utility of the inclusion of a common OCD dimension.

**Methods:**

Participants included 999 individuals participating in the OCD Collaborative Genetics Study (OCGS) and an additional 2363 individuals participating in the OCD Genetic Association Study (OCGAS). We evaluated unidimensional, 5-factor, and hierarchical models of lifetime OCD symptom presentation using confirmatory factor analysis.

**Results:**

Results suggested that the hierarchical model best fit the data. Further evaluation of these models using a Bayesian testlet response model showed that lifetime presence of specific OCD symptoms was differentially associated with lifetime OCD severity. Moreover, symptoms associated with greater lifetime severity were generally reported less frequently than symptoms present at lower levels of lifetime severity. Implications of these findings and future directions are discussed.

## Introduction

Obsessive–compulsive disorder (OCD) is a neuropsychiatric disorder marked by recurrent intrusive thoughts and ritualized behaviors, often aimed at reducing distress.^1^ Specific content of obsessive thoughts and presentation of compulsions can differ considerably between patients.^2^^,^^3^ Even among those with similar phenotypic presentations, variation in symptom presentation over time^4^^–^^7^ and differing motivational factors (eg, harm avoidance vs. incompleteness^8^) can complicate attempts to characterize OCD. Psychometric evaluations of the Yale–Brown Obsessive–Compulsive Scale Symptom Checklist (Y-BOCS-SC^9^)—the gold-standard checklist of OCD symptoms—have typically identified between 3- and 5-symptom domains. Identified symptom domains have included various iterations similar to the following: doubt/checking, contamination, superstitions/rituals, symmetry/hoarding, and taboo thoughts.^10^^–^^14^

Although these symptom domains have provided a useful launching point for clinical researchers attempting to organize and understand the heterogeneity of symptoms reported by patients with OCD (eg,^15^^–^^17^), no firm consensus regarding the underlying structure of OCD symptoms has been reached using these methods.^10^ This may be due, in part, to differences in analytic approaches, including use of exploratory versus confirmatory factor analysis versus principal components analysis^18^^,^^19^ and base unit of analysis—that is, whether studies used item- (symptom) versus category-based analyses. A recent study by Schulze and colleagues^18^ sought to build upon such traditional factor analytic approaches using Bayesian structural equation modeling to examine both first-order (aggressive impulses, sexual obsessions, responsibility, keeping order, magical thinking, cleanliness, somatic obsessions, mental urges, pure repetitions, mental exactness) and second-order symptom dimensions on the Y-BOCS-SC. This study identified 4 second-level factors (incompleteness, taboo thoughts, responsibility, and contamination) consistent with those identified in previous traditional approaches. However, to our knowledge, the existing literature investigating potential symptom subtypes has focused almost exclusively on understanding the structure of OCD symptom dimensions within a short, prescribed time frame (eg, past month), which may preclude insights to be gained by examining cumulative symptom presentation over a patient’s lifetime.

Longitudinal evaluations suggest that OCD symptom presentations are frequently unstable over time, in both childhood^20^ and adulthood,^7^ despite individuals retaining the same OCD diagnosis. There is some suggestion that symptoms often remain within the same domains, even if specific symptoms shift over time^5^; however, studies with a longer follow-up period suggest this may not be the case. Skoog and Skoog^7^ found that more than half of patients exhibited qualitative shifts in symptom expression over a 40-year period. While observed shifts in OCD symptoms presentations over time do not diminish the utility of well-established symptom domains, these shifts may suggest an additional common source of variability in the propensity to experience symptoms across domains that may aid in organizing investigations of the lifetime course of this disorder.

The OCD Collaborative Genetics Study (OCGS) has facilitated heritability analyses of OCD symptom domains. Pinto and colleagues^21^ found that of the 5 identified symptom dimensions (doubt/checking, hoarding, symmetry/ordering, cleaning, and taboo thoughts), all domains, except symmetry/ordering, had significant sibling–sibling associations, with associations being the strongest for hoarding and taboo thoughts. Other investigations have found mixed support for differential familial associations for these 5-symptom domains. Cullen and colleagues^22^ found that no symptom domains were predictive of OCD in first-degree relatives; however, significant sibling–sibling correlations were found for both hoarding and ordering/symmetry domains. Alsobrook and colleagues^23^ also found support for genetic transmission of a symmetry/ordering subtype of OCD. In contrast, van Grootheest et al.^24^ found specific genetic factors related to a contamination dimension, though there were no significant genetic associations with either rumination or checking dimensions. Chacon and colleagues^25^ also found significant sibling–sibling correlations for a contamination dimension, but only when both siblings were male. Finally, Katerberg and colleagues^11^ found that each of 5 factors (taboo, contamination/cleaning, doubts, superstitions/rituals, and symmetry/hoarding) was heritable, though some factors were linked to the same genetic influences (eg, taboo and doubts factors). Additionally, significant sibling–sibling correlations were found for a hoarding dimension, but only when both siblings were female. Thus, while there is some suggestion of the heritability of OCD symptom domains, specific findings are quite mixed.

Beyond individual symptom domains, some investigations have also found support for a broader “OCD” factor. van Grootheest et al.^24^ identified a single higher order “OC behavior” dimension determined to be influenced by genetic factors. Katerberg and colleagues^11^ also found significant heritability for the 5-symptom dimensions as well as for a single underlying dimension of OCD symptoms. Overall, heritability studies support the importance of both specific symptom domains and the heritability of a broad dimension that may underlie variability in lifetime OCD symptoms. In line with this notion, a number of recent studies have considered whether treating OCD as a continuous, underlying dimension (vs. a categorical diagnosis) might have utility in population based, genetics studies, including work on the relationship between genetic factors and OC traits,^26^^,^^27^ and insulin signaling.^28^ To capture this underlying OCD dimension, some studies have relied on a simple summation of number of OCD symptoms as a proxy for OCD severity or on summing Likert-type items rating severity of each symptom assessed; however, underlying assumptions of such an approach (eg, treating each symptom as of equal weight in calculating severity in summing presence vs. absence of symptoms) may limit information to be gleaned. In contrast, a latent variable modeling approach, as we propose here, would allow for variability in strength of relationships between items and their respective underlying factors, which may ultimately serve as a more robust measure of lifetime OCD severity.

Overall, the earlier findings suggest that a focus on identifying specific symptom dimensions or subtypes may tell only part of the story, particularly where OCD symptoms across the lifetime are considered. While previous studies based on the Y-BOCS-SC have organized symptoms using either a single or multidimensional model, to our knowledge, no studies have considered a combined approach to lifetime OCD symptoms in which variability is represented by both an underlying propensity to experience symptoms of OCD and unique domains that further organize symptom expression. Undertaking a reanalysis of data from the OCD Collaborative Genetics Study (OCGS), our primary goal was to evaluate whether a hierarchical structure to lifetime OCD symptom presentations would better fit the data than the 5-factor model previously found by Pinto et al.^21^, allowing separation of an underlying propensity to experience symptoms across domains while also capturing unique sources of variability within each symptom domain. To further evaluate the strength and utility of this model, we next sought to test it in a second sample, derived from the OCD Collaborative Genetics Association Study (OCGAS). Finally, we investigated this hierarchical model as a new method for organizing lifetime OCD symptom presentation using a Bayesian hierarchical item response model to examine the relation between lifetime OCD symptom presentation and OCD severity.

## Methods

### Participants

Participants included 999 individuals participating in the OCD Collaborative Genetics Study (OCGS^29^) and an additional 2363 individuals participating in the OCD Genetic Association Study (OCGAS^30^). For a detailed description of recruitment of the OCGS and OCGAS samples, as well as data collection procedures, refer to Samuels et al.^29^ and Mattheisen et al.^30^ respectively. Briefly, the OCGS study recruited sibling pairs who met criteria for OCD at any time during their lives, their biological parents, and other first- and second-degree relatives with OCD. The OCGAS sample included individuals whose OCD had onset before age 18, as well as parents and unaffected siblings. Written, informed consent (or assent, for children) was obtained from each participant prior to the clinical interview. The OCGS and OCGAS protocols were approved by the local institutional review board at each participating site.

The majority of OCGS participants met criteria for a lifetime DSM-IV diagnosis of OCD (62.5% of the sample). The remainder of the OCGS sample consisted of unaffected relatives. The sample was predominantly Caucasian (97%) and female (62%), with an average age of 41.2 years (SD = 19.5). In the OCGAS sample, 80.3% met criteria for a current DSM-IV diagnosis of OCD. The OCGAS sample was also predominantly Caucasian (90%) and female (58%) with an average age of 33.6 (SD = 17.9).

### Measures

#### Yale–Brown Obsessive–Compulsive Scale Symptom Checklist (Y-BOCS-SC^31^^,^^32^)

The Y-BOCS-SC is a 64-item clinician-administered checklist inquiring about the presence of a range of specific obsessions and compulsions. The presence or absence over the lifetime of each symptom included in the checklist was coded. Additionally, the severity of each symptom during the worst period of OCD was rated on a 4-point Likert-type scale, and age of onset of each symptom was recorded.

#### Yale–Brown Obsessive–Compulsive Scale (Y-BOCS^31^^,^^32^)

The Y-BOCS is a 10-item, clinician-administered scale assessing severity of obsessions and compulsions. This measure was used to assess symptom severity during the *worst* identified period of OCD. Items assessed the amount of time, the extent of interference, degree of distress, the extent of resistance, and degree of control the participant had over their OCD during this time and were rated on a 0 to 4 scale. Previous factor analyses of the 10 clinician ratings have supported either 2 domains reflecting obsession (3 symptoms) and compulsions (3 symptoms), respectively, or a third potential domain reflecting resistance and control of symptoms (4 symptoms), though findings have been mixed. Y-BOCS ratings were not generated for cases with no DSM-IV diagnosis of OCD and no report of OCD symptoms on the Y-BOCS-SC (*n* = 215) and cases were assigned a score of 0. Y-BOCS ratings were not available for *n* = 61 additional cases.

### Analytical procedures

#### Evaluating a primary dimension of OCD

**Figure 1.**
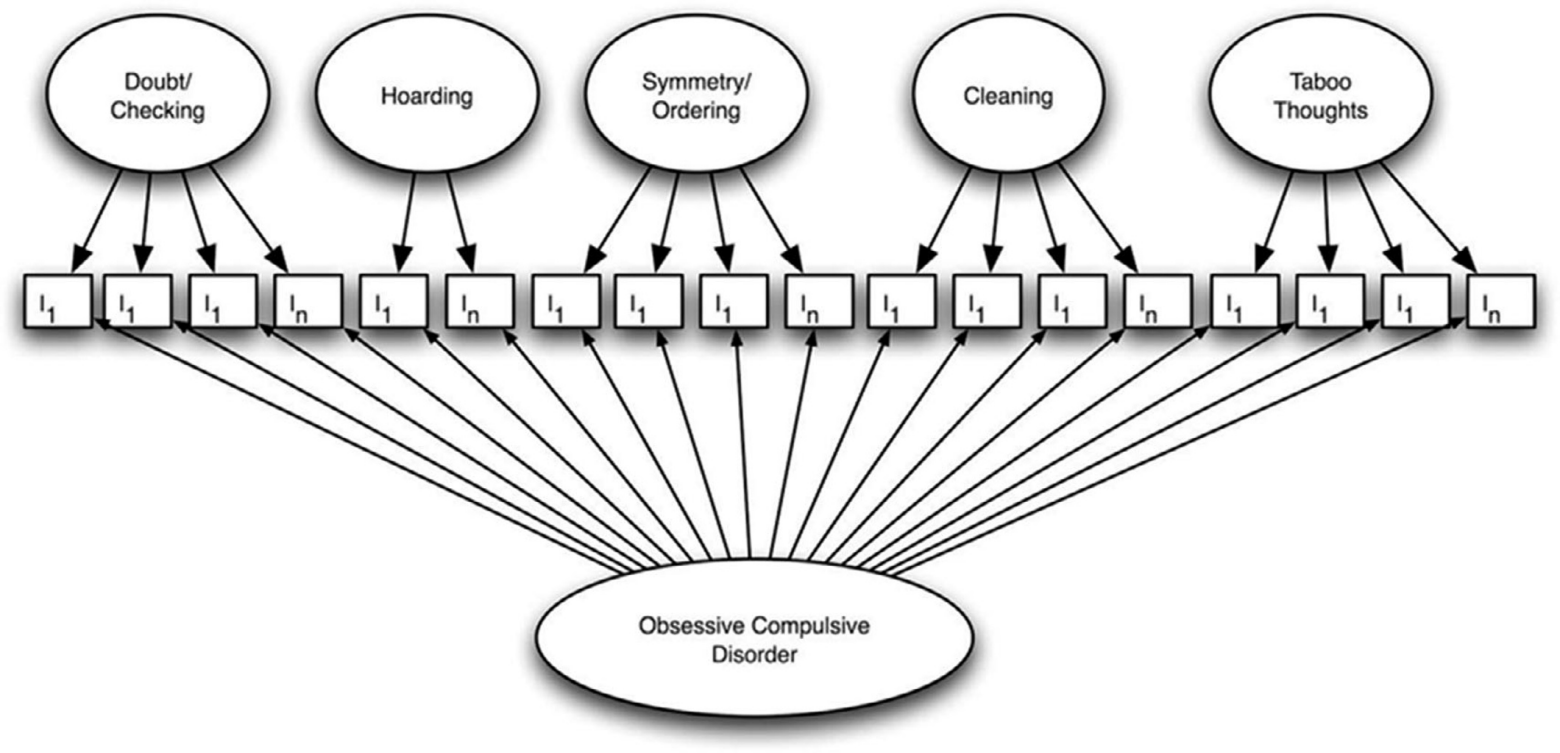
Schematic of planned hierarchical bifactor model for the YBOC-SC. (Not all items or model parameters are explicitly represented.)

In separate sets of analyses within the OCGS and OCGAS samples, confirmatory factor analyses (CFAs) were conducted on the Y-BOCS-SC to evaluate the addition of a unidimensional factor to the 5-factor model found previously in the OCGS. We also conducted a CFA on the Y-BOCS items to evaluate the strength of a primary dimension underlying clinician ratings. Three models were compared to determine which model best fit the data: (1) a unidimensional model in which all checklist items loaded on a single factor, (2) the previously found 5-factor model, which includes doubt/checking, hoarding, symmetry/ordering, cleaning, and taboo thoughts factors, and (3) a hierarchical model including the same 5 factors in model 2, as well as allowing all items to load on a unidimensional factor, as in model 1 (see Figure 1 for schematic). Using similar methods, we evaluated the Y-BOCS ratings comparing a unidimensional, 2-factor multidimensional,^33^ and hierarchical model. All CFA models were conducted with full information maximum likelihood estimation with Metropolis-Hastings Robbins-Monro algorithm (MHRM)^34^ for high-dimensional ordered categorical items using package “mirt” and R statistical analysis software.^35^ Model comparisons were evaluated based on standard fit indices: Akaike information criterion (AIC), Bayesian information criterion (BIC), and chi-square testing of the −2 log likelihood.

#### Testlet response model

In order to examine the relation between the propensity to experience OCD symptoms and the likelihood of reporting each of the individual OCD symptoms, we elected to use a Bayesian hierarchical item response model. Although item response models typically presume the probability of symptom endorsements is due primarily to variability on a single underlying factor—here, OCD—the significant additional variability within each of the 5-symptom domains can be accommodated statistically, by adding a random effect to a standard IRT model that is common to the group of symptoms or “testlets”.^36^^,^^37^ The testlet model (SCORIGHT^38^) we employed uses Bayesian methods^39^ for obtaining estimates of 2 statistical parameters that reflect: (1) the threshold level of lifetime OCD propensity associated with each symptom and (2) how well each symptom discriminates among patients with different levels of lifetime OCD symptoms. The focus of the SCORIGHT method is on generating samples from the posterior distribution of each of the model parameters. This is accomplished by using a Markov chain that repeatedly samples for a parameter until it converges on a particular distribution (Markov chain Monte Carlo: MCMC). The SCORIGHT program requires evidence for convergence of the Gibbs sampler before drawing inferences from the resulting distributions of the model parameters (a = discrimination; b = associated level of OCD) and level of OCD for each respondent (mean = 0; SD = 1). To evaluate convergence, we allowed 40 000 iterations^40^ across 2 separate Markov chains with different starting values, discarded the first 10 000 iterations and compared resulting outputs across each chain using the F-test convergence criterion of less than 1.2 to indicate reasonable convergence.^41^

## Results

### OCD collaborative genetics study (OCGS)

#### Support for a primary dimension

**Figure 2.**
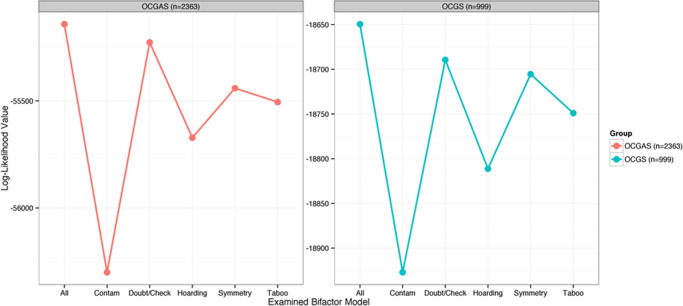
Model fit statistic for hierarchical models from OCGS and OCGAS samples with all 5 domains and models with each domain left out. Higher log-likelihood values indicate weaker model fit and thus the significance of leaving each domain out when describing variability in symptoms beyond that explained by a general OCD dimension.

The 3 models listed earlier were fit to the 64 Y-BOCS-SC items (fit indices listed in Table 1). Comparing fit indices across the 3 models, it is clear that the hierarchical model (Model 3) provided the best fit to the data. This finding suggests that, in addition to variability among Y-BOCS-SC items associated with the 5-symptom domains, lifetime OCD symptom presentation also was strongly related to a common “OCD” factor. We examined the relative strength of relationships between each symptom with the primary symptom domain and with the common OCD dimension. The symptoms from contamination and hoarding domains retained the strongest independent relationship with their respective symptom domain. After adjusting for shared relationships with the primary OCD dimension, all items from the contamination and hoarding domains retained significant loadings on their respective symptom domains (range: 0.35–0.64). In contrast, only 3 of 16 items from doubt/checking, 5 of 15 items from symmetry, and 5 of 18 items from taboo had loadings of this strength. To assess the relative importance of each of the 5 domains, we fit a series of models that evaluated the fit of hierarchical model with and without each of the 5 individual domain scales. Models that included all 5 domains along with a common OCD dimension consistently had the best indicators of model fit. Figure 2 displays the log likelihood value for the series of models including either all 5 domains or with each domain left out, in turn. When compared to the model with all 5 domains along with a common OCD dimension (Model 3), the largest change in model fit was observed when either contamination or hoarding domains were left out. The smallest change in fit was observed when the doubt/checking domain was left out. Inclusion of each domain significantly improved model fit (*p* < 0.001). These results suggest the largest source of additional independent variability beyond that shared with a common dimension of OCD were the contamination and hoarding domains. Based on these findings, we sought to further evaluate the relation between presentation of individual symptoms and this common continuum representing a propensity to experience lifetime OCD symptoms (ie, the common OCD dimension).

**Table 1.** Fit Indices of CFA Models for YBOCS-SC in the OCGS and OCGAS Samples Indices Suggest Significant Improvement of Hierarchical Bifactor Model over Single and Multiple Factor Models

Model	df	AIC	BIC	Log-likelihood	χ^2^	df	*p*
OCGS (*n* = 999)							
Single	591	39 285.37	39 913.43	−19 514.68			
Five-factor	591	40 666.19	41 294.25	−20 205.09	−1380.82	0	1
Bifactor	527	37 683.11	38 625.21	−18 649.56	1730.259	64	0
OCGAS (*n* = 2360)							
Single	1869	116 115.9	116 854.2	−57 929.96			
Five-factor	1869	116 458.2	117 196.4	−58 101.08	−342.231	0	1
Bifactor	1805	110 668.2	111 775.6	−55 142.11	5917.934	64	0

#### Characteristics of symptoms reported on the Y-BOCS-SC

**Figure 3.**
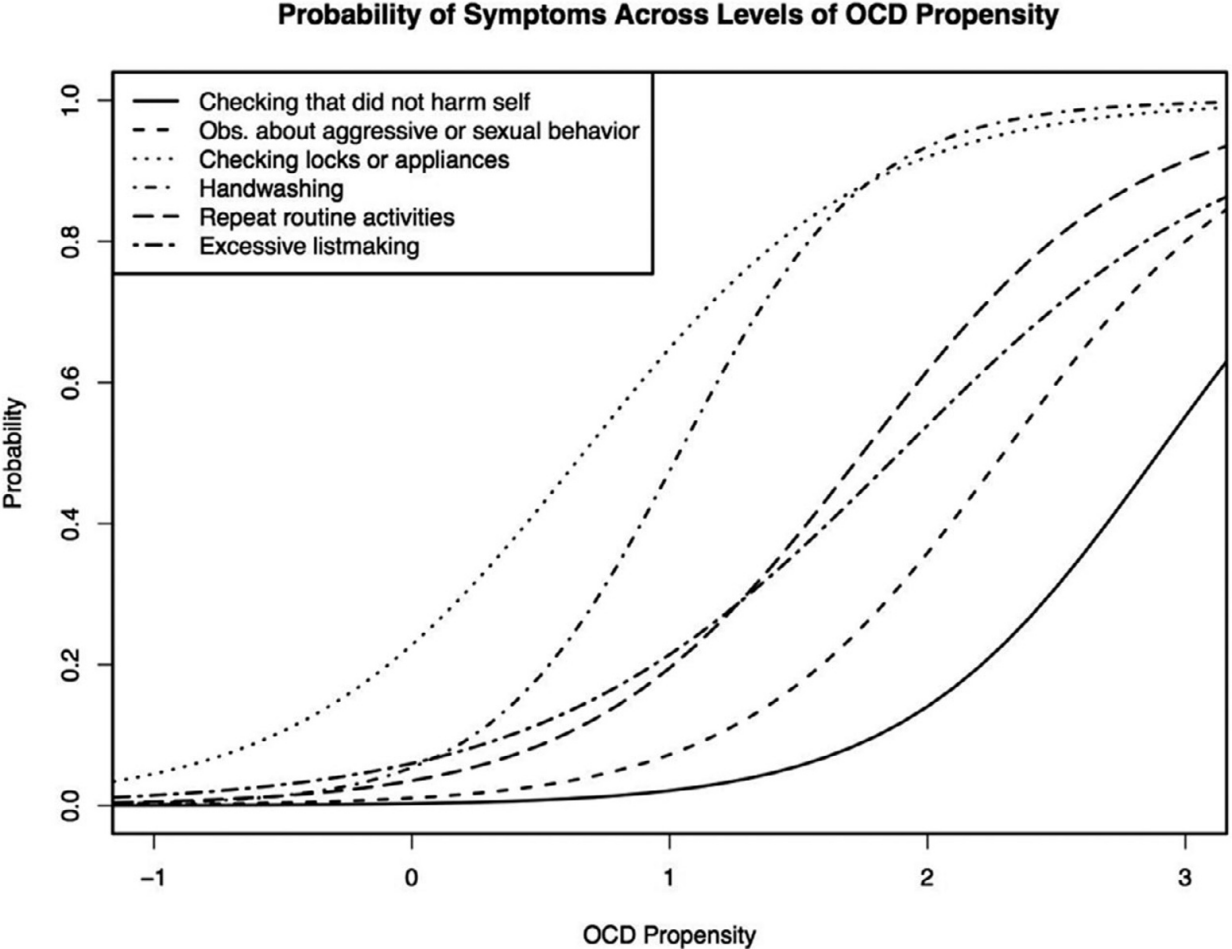
Item characteristic curves, for example, items associated with different degrees of OCD propensity (mean = 0, SD = 1).

We fit the testlet IRT model to the OCGS responses and observed convergence estimates for threshold level (values from 50th, 97.5th quantile = 1.00, 1.01) and discrimination (values from 50th, 97.5th quantile = 1.00, 1.01) parameters were within an acceptable range. Table 2 presents discrimination (a) and difficulty (b) parameters for each Y-BOCS-SC symptom in the OCGS sample. Y-BOCS-SC symptoms were associated with a broad range of OCD (b range = 0.54–2.65). (See Figure 3 for examples of symptoms that provide information about different ranges of OCD.) For example, reports of checking locks or appliances (a = 1.78; b = 0.55) was a common symptom reported by approximately a third of patients in this sample which reflects low levels of OCD and was slightly less discriminating than other symptoms presented here. Reported handwashing was a highly discriminating symptom (a = 2.75; b = 0.85) and was likely to be observed at levels of OCD approximately 1 standard deviation above the average level of OCD in this sample. Review of tabled estimates suggests which symptoms are more or less discriminating and which symptoms are associated with higher and lower levels of lifetime OCD propensity.

**Table 2.** Item Parameter Estimates From Bayesian Testlet Model for OCGS and OCGAS Samples^a^

Item	OCGS (*n* = 999)				OCGAS (*n* = 2363)			
	Slope	SE	Severity	SE	Slope	SE	Severity	SE
Concern with illness	1.83	0.17	1.16	0.08	1.37	0.10	1.28	0.07
Fear of harming self	2.10	0.23	1.42	0.10	1.61	0.11	1.46	0.07
Fear of harming others	1.81	0.18	1.17	0.08	1.79	0.11	1.05	0.05
Fear of harming others if not careful	2.26	0.13	1.19	0.04	1.81	0.12	1.37	0.06
Concern with sacrilege/blasphemy	1.90	0.20	1.56	0.11	1.99	0.17	1.84	0.09
Concern with morality	1.88	0.12	1.27	0.05	1.88	0.12	1.27	0.05
Fear of saying things	2.03	0.26	1.97	0.15	1.84	0.13	1.38	0.06
Fear of not saying right thing	1.89	0.22	1.59	0.13	1.89	0.22	1.59	0.13
Concern with body part	1.35	0.15	1.98	0.17	1.13	0.09	2.37	0.15
Need to tell/ask/confess	1.91	0.18	0.96	0.07	1.65	0.09	0.75	0.04
Fear of losing things	1.81	0.20	1.52	0.11	1.23	0.10	1.84	0.11
Checking if something terrible happened	1.84	0.22	1.67	0.13	1.76	0.13	1.64	0.08
Checking that did not make mistake	2.12	0.19	0.91	0.06	1.51	0.10	1.04	0.05
Nonchecking measures to prevent harm	1.93	0.27	2.20	0.19	1.31	0.11	2.50	0.16
Ritualized eating	1.36	0.14	2.04	0.16	0.98	0.08	2.31	0.16
Checking that did not harm others	1.29	0.13	2.30	0.18	2.06	0.15	1.55	0.07
Concern with bodily waste	2.13	0.21	1.45	0.09	1.86	0.12	1.36	0.06
Concern with dirt/germs	3.24	0.34	0.73	0.05	2.38	0.15	0.67	0.04
Concern with environmental contaminants	2.02	0.23	1.96	0.12	1.39	0.11	2.38	0.12
Concern with household chemicals	2.16	0.26	1.98	0.13	1.42	0.11	2.35	0.11
Concern with animals	2.24	0.26	1.85	0.12	1.39	0.10	2.56	0.12
Bothered by sticky/residues	1.85	0.19	1.81	0.12	1.42	0.10	1.97	0.09
Concern will get ill from contamination	2.11	0.22	1.48	0.09	1.64	0.11	1.54	0.07
Concern will contaminate others	1.99	0.22	1.90	0.13	1.35	0.10	2.26	0.11
Tactile concern	1.75	0.21	2.16	0.15	1.04	0.09	3.01	0.19
Handwashing	2.75	0.26	0.85	0.06	2.41	0.15	0.64	0.04
Showering or grooming	1.69	0.16	1.39	0.10	1.36	0.08	1.09	0.06
Cleaning household items/objects	1.46	0.15	1.58	0.12	1.09	0.08	1.76	0.10
Measures to prevent contaminant contact	1.40	0.17	1.74	0.16	1.52	0.10	1.63	0.08
Hoarding obsessions	1.39	0.16	1.13	0.10	3.35	0.51	1.51	0.09
Hoarding compulsions	1.00	0.10	1.27	0.13	2.37	0.29	1.70	0.10
Fear of responsibility for something terrible	1.71	0.17	1.27	0.09	1.45	0.10	1.12	0.06
Somatic checking	1.50	0.16	1.85	0.14	1.09	0.09	2.25	0.15
Symmetry or exactness with magical thinking	1.49	0.16	1.69	0.13	1.01	0.08	1.99	0.13
Checking locks or appliances	1.78	0.15	0.55	0.06	1.33	0.08	0.46	0.04
Checking that did not harm self	2.15	0.31	2.65	0.23	1.63	0.17	2.94	0.20
Rereading/rewriting	1.98	0.17	0.73	0.06	1.72	0.10	0.45	0.04
Repeat routine activities	1.82	0.20	1.66	0.13	1.56	0.10	1.29	0.06
Counting compulsions	1.77	0.16	1.05	0.07	1.45	0.09	0.85	0.05
Ordering compulsions	1.54	0.13	0.54	0.06	1.13	0.07	0.57	0.05
Mental rituals	1.50	0.16	1.64	0.13	1.23	0.09	1.59	0.09
Need to touch	1.63	0.16	1.19	0.09	1.39	0.08	1.16	0.06
Blinking or staring rituals	1.94	0.23	1.81	0.13	1.51	0.12	1.99	0.10
Symmetry or exactness (w/out magical thinking	1.21	0.12	1.29	0.11	1.01	0.07	1.15	0.07
Need to know	1.69	0.17	1.39	0.10	1.34	0.09	1.28	0.07
Excessive list making	1.39	0.16	1.82	0.15	1.05	0.08	1.98	0.11
Violent images	2.20	0.23	1.19	0.07	1.93	0.12	1.08	0.05
Fear of blurting out	1.89	0.22	1.77	0.13	2.17	0.16	1.50	0.06
Fear of doing something embarrassing	1.97	0.23	1.87	0.14	1.84	0.14	1.63	0.07
Fear acting on unwanted impulse	1.93	0.21	1.57	0.12	2.02	0.13	1.42	0.06
Forbidden sexual thoughts	2.03	0.23	1.47	0.11	1.82	0.12	1.54	0.07
Obsessions involving incest	2.38	0.35	1.93	0.15	2.20	0.17	1.73	0.07
Obsessions about sexuality	2.03	0.25	1.91	0.14	1.63	0.12	1.84	0.09
Obsessions about aggressive sexual behavior	2.08	0.28	2.06	0.17	1.86	0.17	2.14	0.12
Fear of stealing	1.79	0.23	2.43	0.21	1.90	0.16	2.15	0.11
Intrusive nonviolent images	1.80	0.25	2.28	0.20	1.65	0.14	2.29	0.13
Intrusive nonsense sounds	1.35	0.14	1.78	0.15	1.19	0.09	1.98	0.12
Bothered by noises	1.59	0.19	2.09	0.17	1.22	0.10	2.19	0.14
Lucky/unlucky numbers	1.78	0.19	1.51	0.11	1.41	0.09	1.28	0.06
Special colors have significance	2.03	0.28	2.25	0.20	1.38	0.11	2.51	0.15
Superstitious fears	1.73	0.18	1.49	0.10	1.34	0.10	1.84	0.10
Superstitious behaviors	1.51	0.16	1.70	0.14	1.07	0.09	2.35	0.16
Trichotillomania	1.30	0.16	2.37	0.22	0.96	0.09	3.11	0.23
Self-mutilation	1.13	0.13	2.35	0.22	0.92	0.09	3.00	0.25

a Slope reflects the discrimination parameter (a). Severity reflects the difficulty parameter (b).

Among the 75% of patients who reported at least 1 of the 64 Y-BOCS-SC symptoms, we observed few common patterns of symptom presentation within those reporting similar total numbers of symptoms. The 2 most prevalent patterns represented 9 patients who reported a “fear that they had harmed others” as the only Y-BOCS-SC symptom and 8 patients who reported hoarding obsessions and hoarding compulsions. Seventy-one percent of patients reported unique patterns of lifetime symptoms suggesting a high degree of heterogeneity.

#### Characteristics of clinician ratings using the Y-BOCS severity scale

CFA of the Y-BOCS severity scale items also supported the strength of a single primary dimension underlying clinician ratings (loadings range = 0.79–0.95). Table 3 presents item response model parameters for each of the 10 Y-BOCS clinician ratings. The 5-level rating options results in a single estimate of discrimination and 4 estimates to mark the levels of OCD severity associated with each rating level (0–4). Ratings of time occupied with obsessions (a = 3.19; SE = 0.20) and compulsions (a = 2.94; SE = 0.19), and distress associated with obsessions (a = 3.10; SE = 0.22) were the most discriminating ratings. Efforts to resist obsessions (a = 1.59; SE = 0.10) and compulsions (a = 1.42; SE = 0.08) were the least strongly related to overall clinician ratings of OCD severity. Time spent on obsessions (b1–4 = −0.52, 1.73, 3.20, 4.46) and compulsions (b1 = −0.62, 2.01, 3.66, 5.25) each provided ratings of the highest levels of OCD severity. Interference in functioning from obsessions (b1–4 = −0.09, 1.02, 2.28, 3.92) and from compulsions (b1–4 = −0.16, 1.20, 2.56, 4.25) were likely to be observed at similar levels of OCD severity. Distress from obsessions and anxiety if prevented from engaging in compulsions also largely mirrored each other. Degree of efforts to resist obsessions (b1 = −0.21, 0.59, 1.59, 2.15) and compulsions (b1–4 = −0.53, 0.51, 1.50, 2.02) and degree of control over obsession and compulsions were observed at similar levels of OCD severity.

**Table 3.** Item Response Model Parameters for Y-BOCS Severity Scale Clinician Ratings in the OCGS Sample

Y-BOCS item	Mean	SD	a	SE_a_	b_1_	SE_b1_	b_2_	SE_b2_	b_3_	SE_b3_	b_4_	SE_b4_
Obs: Time/frequency	1.92	1.54	3.19	0.20	−0.52	0.06	1.73	0.15	3.20	0.20	4.46	0.24
Obs: Interference	1.40	1.36	2.53	0.17	−0.09	0.05	1.02	0.09	2.28	0.13	3.92	0.18
Obs: Distress	1.89	1.51	3.10	0.22	−0.38	0.05	0.93	0.10	2.40	0.15	4.37	0.23
Obs: Effort to resist	1.58	1.49	1.59	0.10	−0.21	0.06	0.59	0.06	1.59	0.08	2.15	0.10
Obs: Control	2.11	1.62	2.50	0.19	−0.38	0.06	0.54	0.08	1.44	0.12	2.83	0.16
Comp: Time/frequency	1.74	1.37	2.94	0.19	−0.62	0.06	2.01	0.15	3.66	0.20	5.25	0.26
Comp: Interference	1.35	1.29	2.47	0.15	−0.16	0.05	1.20	0.08	2.56	0.11	4.25	0.17
Comp: Distress	1.97	1.48	2.38	0.13	−0.55	0.06	0.92	0.10	2.13	0.13	3.79	0.17
Comp: Effort to resist	1.95	1.56	1.42	0.08	−0.53	0.06	0.51	0.05	1.50	0.07	2.02	0.08
Comp: Control	2.17	1.56	2.09	0.13	−0.58	0.06	0.52	0.08	1.48	0.11	2.93	0.14

Abbreviations: Obs, obsessions, Comp, compulsions.

#### Distribution of OCD symptoms and severity of impairment

**Figure 4.**
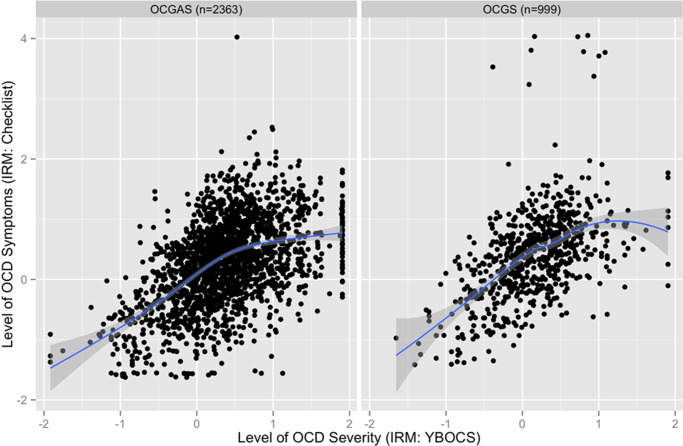
Bivariate relationship between clinician rated impairment on the YBOCS with item response model estimates of OCD checklist severity score and loess smoothed regression line (*r* = 0.74, *p* < 0.01).

The testlet model also generated posterior samples of the estimated level of OCD for each respondent. These samples allow a single point estimate of OCD propensity for each individual as well as standard errors to gauge the relative precision of scores. The precision of estimating individual levels of OCD depends on the match between the propensity for OCD and the relative probability of observing each Y-BOCS-SC symptom. As shown in Figure 4, the Y-BOCS-SC sorts individuals with greatest precision when levels of lifetime OCD symptoms are high (0.5–2 SD of sample mean). Individuals who do not endorse any symptoms cannot be rank ordered further, while several symptoms are available to rank order individuals with more symptoms.

#### Relationship between lifetime OCD symptom reports and clinician-rated maximum lifetime severity

Model-derived OCD lifetime symptom scores maintained rank order of participants’ raw Y-BOC-SC scores (Spearman *r* = 0.97) and showed a strong correlation with Y-BOCS severity scores (Spearman *r* = 0.80, *p* < 0.001; see Figure 4), supporting our interpretation of this unidimensional factor as a metric of lifetime OCD symptoms with strong ties to overall impairment from OCD symptoms. We display a locally weighted regression line to highlight variability in the strength of relationship between lifetime symptom reports and clinician ratings of impairment. Strong linear relationships of impairment ratings and symptom reports were observed through the bulk of the distribution of scores with weakened relationships in the highest range of symptoms (>1 SD above the mean).

**Figure 5.**
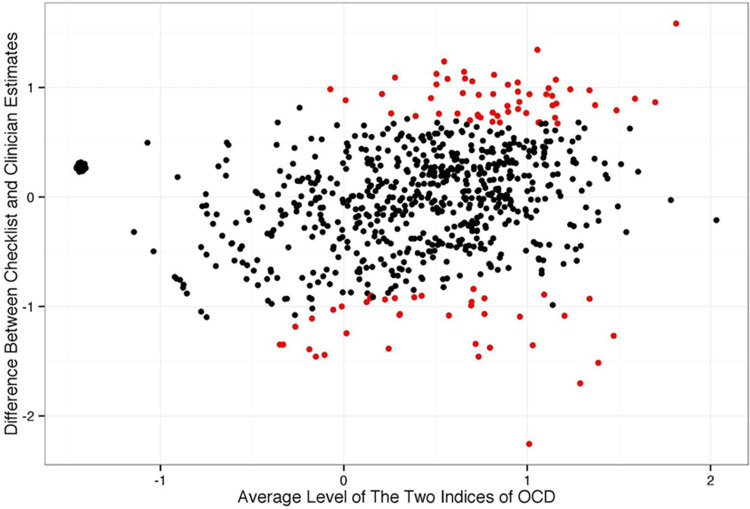
Bland–Altman plot to assess agreement between Y-BOCS-SC and Y-BOCS ratings of OCD severity. Each point reflects the difference between measures of posterior estimated level of OCD severity (Y-BOCS-SC minus Y-BOCS). Patients with large disagreement (falling outside 95% confidence intervals) between the Y-BOCS-SC and Y-BOCS are colored red.

With support for an association between propensity for lifetime symptoms and clinician rated impairment, we computed rates of agreement about each patient level of OCD using the 2 methods and constructed a Bland–Altman plot. With no gold-standard criterion, we used a common mean of the 2 estimates and the joint standard error of each measure estimate (sqrt[SE^2^_Y-BOCS-SC_ + SE^2^_Y-BOCS_]) to plot the differences between each of the measure estimates (Estimate_Y-BOCS-SC_ − Estimate_Y-BOCS_). Figure 5 shows that the 2 measures’ estimates agreed within expected confidence regions in 90.1% of patients. The largest disagreements occur among patients reporting symptoms and receiving low ratings of severity (red points at the top of the plot). Disagreements were more frequent at the higher range of OCD.

### OCD genetic association study (OCGAS)

#### Support for a primary dimension

As before, the 3 models listed earlier were fit to the 64 Y-BOCS-SC items (fit indices listed in Table 1). Comparing fit indices across the 3 models in this new sample also supported the hierarchical model (Model 3) as providing the best fit to the data. After adjusting for shared relationships with a primary OCD dimension, symptoms from contamination and hoarding domains retained significant loadings (range: 0.43–0.87). In contrast, only 2 of 16 items from doubt/checking, 7 of 15 items from symmetry, and 5 of 18 items from taboo had loadings of this strength. When we compared the relative contribution of each of the 5 subdomains, we again found that the largest source of additional independent variability beyond that shared with a common dimension of OCD was observed for contamination and hoarding domains (see Figure 2). These results support the hierarchical representation of a primary dimension of OCD along with significant independent contributions from each of the 5 subdomains.

#### Characteristics of symptoms from the Y-BOCS-SC

We fit the testlet IRT model to the OCGAS responses and observed convergence estimates for threshold level (values from 50th, 97.5th quantile = 1.00, 1.01) and discrimination (values from 50th, 97.5th quantile = 1.00, 1.01) parameters were within an acceptable range. Table 2 presents results from the testlet IRT model estimation of discrimination and difficulty parameters for each Y-BOCS-SC symptom in the OCGAS sample. The relative rank of symptom severity (“b”) across the OCGS and OCGAS samples was largely maintained with a Spearman rank correlation of 0.87.

#### Characteristics of clinician ratings using the Y-BOCS

CFA of the Y-BOCS items again supported the strength of a single primary dimension underlying clinician ratings (loadings range = 0.65–0.92). We fit the graded response model to the 10 Y-BOCS severity scale items and computed posterior scores for each participant reflecting levels of clinician-rated symptom severity. Similar to findings in the OCGS sample, Figure 4 shows correspondence between posterior scores participant reports on the checklist and posterior scores reflecting clinician ratings.

## Discussion

Our primary goal was to investigate a hierarchical model of lifetime OCD symptom presentation, accounting for both clinically noted heterogeneity in symptom presentation (symptom domains) and homogeneity of the diagnosis (a common OCD dimension). After comparing unidimensional, 5-factor, and hierarchical models, we found that the hierarchical model best fit the data. This supports the notion that a symptom subtype dimensional approach only partially accounts for the vast complexity underlying the disorder, particularly over the lifetime. A common OCD dimension (ie, the unidimensional factor) may, in fact, be useful in disentangling the gamut of symptom subtypes in a lifetime OCD sample. The strong correlation between the common OCD dimension and total scores on the Y-BOCS severity scale suggests that this factor represents the degree of severity of OCD over the lifetime.

After establishing the hierarchical model, we then used a Bayesian hierarchical item-response theory model to investigate the relationship between presence or absence of specific OCD symptoms over the lifetime and OCD severity. While there was significant heterogeneity in observed patterns of lifetime symptoms, we detected several notable findings. First, lifetime presence of specific OCD symptoms was found to be differentially associated with OCD severity. For example, checking to make sure one did not harm oneself is a symptom found more likely to be associated with very severe OCD. However, hand-washing or cleaning rituals are seen with less severe lifetime OCD (though, of course, can also be present in more severe cases, as well). Consistent with this finding, symptoms selectively associated with greater lifetime severity generally were reported less frequently than those symptoms present at lower levels of lifetime severity. These findings suggest that a simple sum of number of lifetime symptoms experienced may not adequately capture illness severity, as the presence of one “high severity” symptom may indeed reflect greater illness severity than the presence of several symptoms typically seen at lower severity levels. These results may also explain inconsistent findings in the literature regarding clinical correlates of symptom subtypes or dimensions. For example, some studies have found that religious and sexual obsessions respond less well to behavior therapy,^5^^,^^42^ where others have not found this difference.^43^^,^^44^ However, our study found specific obsessions within this same category to be associated with different levels of lifetime severity. For example, obsessions regarding aggressive sexual behavior were associated with higher lifetime OCD severity than forbidden sexual thoughts. Thus, while symptoms within respective domains may share some commonalities, these categories seem unlikely to be instructive as to lifetime severity of illness. That is, considering symptom dimension alone, without regard for lifetime severity, may provide an incomplete picture.

In the hierarchical model, we found that symptoms in the contamination and hoarding domains retained the strongest independent relationship with their respective factors. Additionally, we found handwashing to be a highly discriminating symptom. These findings are largely consistent with previous research suggesting that while contamination related obsessions and compulsions are characteristic of one of the most prevalent presentations of OCD,^45^ they are also often resistant to treatment.^46^ Moreover, it is unsurprising that items in the hoarding domain also retained strong independent relations with their respective symptom domain given the historically inconsistent relationship between hoarding and OCD,^47^^–^^49^ with hoarding disorder now recognized as distinct from OCD.^1^ In contrast to the hoarding and contamination domains, removal of the doubt/checking domain revealed more minimal changes to model fit. The field has often struggled to disentangle doubt and checking in individuals with OCD and may confound these symptoms.^44^ Indeed, it is likely that doubt/urge to check about potential harm may underlie many OC-related behaviors.^50^

While our work focused on examining the factor structure of OCD symptoms over the lifetime, our findings were relatively consistent with work by Olatunji and colleagues,^51^ testing a bifactor model of OCD symptom subtypes using the Dimensional Obsessive–Compulsive Scale (DOCS^52^). Results showed improved fit for a bifactor model, defined as a general OC symptom factor alongside the 4-symptom dimensions (ie, contamination, responsibility for harm, unacceptable thoughts, symmetry or ordering) when compared to the 4-factor model, alone. This suggests that a common “OCD” dimension may have utility beyond traditional symptom domains across measures of OCD symptoms and that a common dimension may provide information both within current episode (as in^51^), as well as when considering lifetime symptom presentation, as we have here.

Our findings expand on those of previous genetics investigations in OCD. Within the OCGS sample (used here), Pinto and colleagues^21^ found support for a 5-factor model of OCD symptom dimensions in the Y-BOCS-SC with 4 of the 5 factors showing significant sib–sib associations. Similarly, heritability studies by van Grootheest et al.^24^ and Katerberg et al.^11^ have shown shared variations in OC symptom dimensions with latent common factors. Taken together with findings of this study, these studies suggest inclusion of a common OCD factor in modeling OCD symptom presentations as an important source of information and variability in modeling OCD presentation, particularly across the lifespan, implications of which may be of particular importance to genetic research in OCD. Benefits of using an approach like the one used here include the ability to estimate of individual levels of OCD that includes information about patterns of symptoms endorsed, as well as a measure of uncertainty for individuals indicating whether the pattern of symptoms endorsed is more or less typical. Notably, use of a common dimension, underlying lifetime symptom reports, may represent an alternative method of organizing information on phenotypic expression, which may be of use in genetic, and other, investigations of OCD pathophysiology.

This study has a number of limitations which should be acknowledged. First, the OCGS sample used in this study partially overlaps with the samples used by Hasler et al.^53^ and Pinto et al.^21^ Thus, while this study uses confirmatory factor analytic methods (eg, CFA), these findings must be regarded as exploratory. To partially mitigate this limitation, we confirmed these findings by testing the hierarchical model in the second OCGAS sample. Second, given that the focus of this study was on lifetime symptom presentation and severity, it is unknown whether our findings are generalizable to investigations of current OCD symptom presentations. Because OCD is a chronic disorder, individuals with a prior history of OCD may still experience residual symptoms or relapse.^54^ As such, future studies may consider replicating these findings with individuals with current symptoms and/or analog samples to understand the pattern of symptom domains and the common OCD factor in a broad sample of individuals with OCD assessed with the Y-BOCS-SC. Third, we used the Y-BOCS-SC to evaluate symptom domains given its wide use among clinicians. However, other measures evaluating OC symptom domains and severity may produce different results. Our findings appear consistent with research from Olatunji et al.^51^ testing a bifactor model of the DOCS; however, replication and additional research using other measures is needed. Fourth, the majority of our sample was White and, as a result, findings may not generalize to other races and ethnicities. Future work will benefit from including individuals from diverse backgrounds including race/ethnicity, gender, and sociodemographic status, which may provide us with information about additional factors that may influence symptom presentation. Finally, collection of lifetime symptom data was done retrospectively, in line with study protocols, which may be subject to recall bias. Future studies may employ collateral reports to support one’s history of lifetime symptoms.

Overall, our findings suggest that in characterizing lifetime OCD symptom presentation, inclusion of both a common factor as well as specific symptom domains may provide greater accuracy in disentangling heterogeneous OC symptoms. The addition of a common OCD factor to models of traditional symptom domains has utility in explaining additional variability in symptom presentation and severity of illness. We believe this work may be useful for future genetic studies that incorporate behavioral components and phenotyping. Moreover, these findings suggest a unique relationship between specific OCD symptoms and lifetime severity of illness which ultimately offer a useful tool to clinicians in assessing lifetime severity of illness.

## Data Availability

The data that support the findings of this study were generated as part of the OCD Collaborative Genetics Association Study (OCGAS) consortium. Access to these data are restricted due to the terms of the consortium’s data-sharing agreement, which governs the handling of sensitive genetic and clinical information. Consistent with the consortium’s policy, access to the data can be obtained through a formal request process managed by the consortium’s data access committee. Interested researchers may contact the OCGAS Data Access Committee for information on how to apply for access to the data, subject to review, and approval.
